# Activity in the Fronto-Parietal and Visual Cortex Is Modulated by Feature-Based Attentional Weighting

**DOI:** 10.3389/fnins.2022.838683

**Published:** 2022-04-25

**Authors:** Armien Lanssens, Dante Mantini, Hans Op de Beeck, Celine R. Gillebert

**Affiliations:** ^1^Department of Brain and Cognition, KU Leuven, Leuven, Belgium; ^2^Leuven Brain Institute (LBI), KU Leuven, Leuven, Belgium; ^3^Research Center for Motor Control and Neuroplasticity, KU Leuven, Leuven, Belgium; ^4^Brain Imaging and Neural Dynamics Research Group, IRCCS San Camillo Hospital, Venice, Italy

**Keywords:** fMRI, selective attention, feature-based attentional weighting, dorsal attention network, visual cortex

## Abstract

In day-to-day dynamic activities where sensory input is abundant, stimulus representations in the visual cortex are modulated based on their attentional priority. Several studies have established the top-down role of a fronto-parietal dorsal attention network in selective attention. In the current study, we aimed to investigate whether activity of subregions of this network and the visual cortex is modulated by feature-based attentional weighting, and if so, whether their timecourses of activity are correlated. To this end, we analyzed fMRI data of 28 healthy subjects, who performed a feature-based go/no-go task. Participants had to attend to one or two colored streams of sinusoidal gratings and respond to each grating in the task-relevant stream(s) except to a single non-target grating. Univariate and multivariate fMRI results indicated that activity in bilateral fronto-parietal (frontal eye fields, intraparietal sulcus and superior parietal lobe) and visual (V1–V4, lateral occipital cortex and fusiform gyrus) regions was modulated by selecting one instead of attending to two gratings. Functional connectivity was not significantly different between fronto-parietal and visual regions when attending to one as opposed to two gratings. Our study demonstrates that activity in subregions of both the fronto-parietal and visual cortex is modified by feature-based attentional weighting.

## Introduction

Since the limited capacity of the brain impedes us to process all sensory stimuli at once, selective attention is a crucial ability in daily life (Broadbent, 1957; Lennie, 2003). Depending on the behavioral goal in a certain situation, attentional selection in the visual domain can be endogenously deployed based on spatial locations (e.g., Posner, 1980), objects (e.g., Duncan, 1984; Scholl, 2001) and even features of objects (e.g., Driver and Baylis, 1989; Egeth and Yantis, 1997). For instance, when searching for an item in a cluttered visual scene (e.g., car keys in the kitchen), spatial-based attentional weighting is useful when prior knowledge is available on the location of the task-relevant item (e.g., the kitchen table). Then, information processing is facilitated in that spatial location to allow rapid identification of the item. However, without such prior knowledge, feature-based attentional weighting is a more efficient mechanism to find the task-relevant item, facilitating the processing of items with a particular feature (e.g., the red color of a key chain) throughout the visual field based on an internal representation of what the item looks like.

In order to resolve the competition of sensory input for processing resources, it has been established that selective attention modifies stimulus representations in the visual cortex based on behavioral relevance (Kastner and Ungerleider, 2000; Maunsell and Treue, 2006). As a result, activity levels in (neuronal subpopulations of) visual areas are biased for attended over unattended spatial locations (Moran and Desimone, 1985; Luck et al., 1997; Kastner et al., 1999; Reynolds et al., 1999; McMains and Somers, 2004), for attended over unattended features (e.g., motion) (Zanto et al., 2010; Zhang et al., 2018) and even for relevant feature values (e.g., specific direction of motion) (Kamitani and Tong, 2006; Liu et al., 2007, 2011; Serences et al., 2009). Here, top-down processes appear to enhance visual units that process relevant locations or features, while neurons processing other (irrelevant) locations or features are suppressed (Smith et al., 2000; Slotnick et al., 2003; Gazzaley et al., 2005, 2008; Chang and Egeth, 2019). These relevance-based modulations of feature representations are not restricted to the attended location, but actually spread throughout the entire visual field (Saenz et al., 2002; Martinez-Trujillo and Treue, 2004; Bichot et al., 2005; Serences and Boynton, 2007). Thus, selective attention allows us to overcome our limited information processing capacity by enhancing visual units that process relevant stimulus properties, even outside the focus of attention when selecting information based on features, while suppressing other visual units.

Several studies have investigated the origin of top-down signals to the visual cortex to gain insight in the process of selective attention. The fronto-parietal dorsal attention network (DAN), encompassing the frontal eye fields (FEF), intraparietal sulcus (IPS) and superior parietal lobe (SPL), has been reported to play a key role in each mechanism of selective attention: spatial-based (Nobre et al., 1997; Gitelman et al., 1999; Gillebert et al., 2012a), object-based (Serences et al., 2004; Baldauf and Desimone, 2014) and feature-based (Shulman et al., 1999; Lanssens et al., 2020). Importantly, the DAN has been shown to contain topographic maps encoding the attentional priority of items of the visual field, in which each stimulus is weighted by its respective bottom-up (e.g., physical salience) and top-down (e.g., behavioral relevance) value (Silver and Kastner, 2009; Bisley and Goldberg, 2010). It has been suggested that the DAN drives the process of selective attention by modifying stimulus representations in the visual cortex based on their respective priority in the topographic map (Corbetta and Shulman, 2002). Multiple studies involving spatial-based attentional weighting obtained results in favor of top-down signals from regions in the DAN to the visual cortex, congruent with feedback based on a highlighted location in the topographic map (Kastner et al., 1999; Bressler et al., 2008; Gregoriou et al., 2009; Lauritzen et al., 2009; Gillebert et al., 2012a; Liu et al., 2016; Wiesman et al., 2018). These findings are supported by evidence demonstrating the presence of white matter connections between subregions of the DAN and visual regions (Greenberg et al., 2012). Considering that a similar neural basis for spatial and non-spatial mechanisms of selective attention has been suggested (Wojciulik and Kanwisher, 1999; Giesbrecht et al., 2003; Greenberg et al., 2010; Cohen and Maunsell, 2011; Liu and Hou, 2013), the DAN may similarly use information on features to modify stimulus representations in visual regions (Giesbrecht et al., 2003; Egner et al., 2008). However, study-based evidence in the context of feature-based attentional weighting is still limited. Multiple studies used multivariate analysis techniques to demonstrate that attended over unattended features can not only be distinguished in visual regions, but also in regions of the DAN (Liu et al., 2011; Ibos and Freedman, 2014; Ester et al., 2016; Reeder et al., 2017; Sapountzis et al., 2018). Congruently, a more recent study provided a link between activity in parietal regions of the DAN and the correct discrimination of relevant and irrelevant information based on features, with TMS disrupting task performance (Jigo et al., 2018). Taken together, the DAN appears to contain stimulus representations that are modulated by feature-based attentional weighting, but the interaction between the DAN and visual regions is still unclear. To this end, Zanto et al. (2010) used an fMRI functional connectivity analysis to investigate top-down signals to the visual cortex when behavioral relevance was defined in feature space (color or motion, respectively). Results suggested a prominent role for the prefrontal cortex (specifically, the inferior frontal junction) [also see Zanto et al. (2011)], while no evidence was found for the involvement of regions in the parietal cortex in color modulation. However, Zanto et al. (2011) used a working memory paradigm which might have led to the strong involvement of prefrontal regions (D’Esposito et al., 2000; Barbey et al., 2013). Thus, further research on feature-based attentional weighting in the DAN and the subsequent interaction between the DAN and the visual cortex is valuable.

In the current study, we aimed to investigate whether activity in subregions of the DAN and the visual cortex is modulated by feature-based attentional weighting, and if so, if the respective timecourses of activity suggest an interaction between fronto-parietal and visual regions. To this end, participants performed a go/no-go task under fMRI, in which two differentially colored streams of stimuli were displayed. Here, different task conditions required participants to either select one stream or attend to both streams. Task conditions were sensorially matched and only differed in the need to prioritize information based on one or two colors. The task of the current study is different from those in previous feature-based attention studies (e.g., Desimone and Duncan, 1995) in that it is more dynamic and has a sustained attention baseline against which attentional effects in the conditions of interest can be evaluated. Functional neuroimaging data were analyzed by using a combination of univariate, multivariate and functional connectivity analysis techniques. Considering the differences in attentional priority between task conditions, we expected that attending to one as opposed to two streams of stimuli would modify activity in subregions of the DAN. Similarly, we hypothesized that activity patterns in the visual cortex would be modulated by attention, and that the timecourses of activity in subregions of the DAN would correlate with those of subregions of the visual cortex.

## Methods

### Participants

Twenty-eight healthy volunteers (22 female, 18–29 years, all right-handed) participated in the current study. All participants had normal or corrected-to-normal vision and all of them reported not to suffer from color blindness. The study was approved by the Ethics Committee Research UZ/KU Leuven (Reference number: S60929). All participants gave written informed consent in accordance with the Declaration of Helsinki.

### Task and Stimuli

#### Go/No-Go Task

**FIGURE 1:**
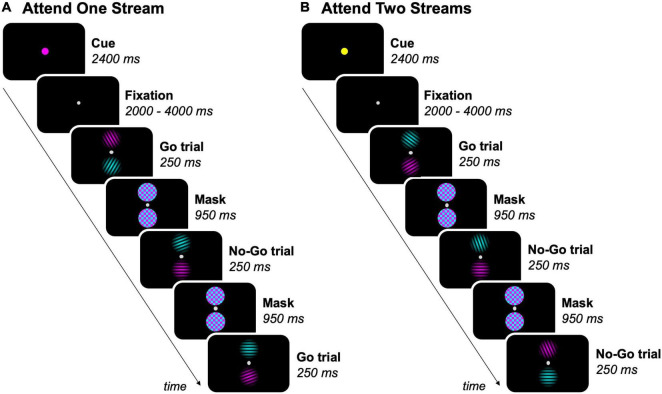
Feature-based go/no-go task. Experimental blocks either required participants to **(A)** attend to one stream (magenta, as shown, or cyan), or **(B)** attend to two streams of gratings. The task-relevant streams was/were indicated by a colored dot before each block. As an example, 3 trials per block are shown, but the total amount of trials per block was 18. Participants were instructed to press a button on a response box when a target (grating ≠ 90°) was present in the task-relevant streams (= go trials), but had to inhibit the response when a non-target (grating = 90°) was present in a task-relevant stream (= no-go trials).

Stimulus presentation and response registration were controlled by a personal computer running Presentation 19.0 (Neurobehavioral Systems, Berkeley, CA, United States). Participants performed a feature-based go/no-go task in the MR scanner (Figure 1). The task was presented to participants by means of a projector (resolution: 1,920 × 1,080 pixels, refresh rate: 60 Hz; NEC Display Solutions, IL, United States), in which the projection of stimuli on a white screen was visible through a mirror mounted on the head coil. In each task trial, two equiluminant streams of circular sinusoidal gratings (size = ∼1.50° visual angle, spatial frequency = 0.07 cycles/px) were displayed on the vertical meridian, separated by a fixation dot (size = ∼0.15° visual angle, offset from stimuli = ∼0.80° visual angle), on a black screen. One stream was colored in cyan and the other one in magenta. The magenta and cyan stream of gratings randomly switched location between trials. This resulted in half of the trials within each block where the magenta stream was above the fixation dot and the cyan stream below, and the other way around for the other half of the trials. Each pair of gratings was presented for 250 ms and was followed by a mask for 950 ms, which consisted of two cyan and magenta colored noise patches (size = ∼1.60° visual angle).

The feature-based go/no-go task had a mixed experimental design, with each participant completing nine fMRI runs of nine experimental blocks each. Experimental blocks began by presenting a cue for 2,400 ms, indicating the task-relevant streams in the upcoming block, followed by a jittered fixation interval between 2 and 4 s. The cue was a fixation dot that doubled in size and had one out of three possible colors: cyan or magenta (one of both streams task-relevant), or yellow (both streams task-relevant). Each sequence of three blocks contained each of these conditions exactly once in a fixed order within runs, with participants being informed about their order prior to each run. In experimental blocks where one stream of gratings was task-relevant, participants were instructed to attend to the cued (= task-relevant) stream and ignore the uncued (= task-irrelevant) stream. In experimental blocks where both streams of gratings were task-relevant, the two streams had to be attended simultaneously. The order of the blocks was counterbalanced across runs and participants. After each block, a fixation period of 6 s occurred.

Each experimental block consisted of 18 task trials, resulting in a total of 162 trials per run. Gratings had nine possible orientations ranging from 10° to 170° with a 20° interval. In each sequence of nine trials, each stream contained each of nine possible orientations exactly once in random order, with the restriction that co-presented gratings were never the same. Participants were instructed to fixate the center of the screen and press a button on the response box with their right thumb every time a target (grating ≠ 90°) was present in the task-relevant stream(s) (= go trials). However, participants had to inhibit the response when a non-target (grating = 90°) was present in a task-relevant stream (= no-go trials). Participants were asked to prioritize accuracy over speed while performing the task. The number of no-go trials was matched for blocks where either one or two streams of gratings required attention by presenting a grating with a random target orientation instead of the grating with the non-target orientation for each sequence of nine trials of one stream in blocks where both streams were task-relevant.

#### Meridian Mapping

Stimulus presentation and response registration were controlled by a personal computer running the Psychophysics Toolbox 3.0.11 in MATLAB (MATLAB 8.5, The MathWorks Inc., Natick, MA, United States). Following a standard procedure from Tootell et al. (1995), meridian mapping runs were administered to delineate visual cortical regions. Checkerboard wedges consisting of different shapes (with varying numbers, sizes and colors) were presented for 250 ms with an inter-stimulus interval of 125 ms. Experimental blocks presented these wedges either horizontally or vertically for 15 s. Each succession of one horizontal and one vertical block was followed by a fixation block of 15 s. This procedure was repeated eight times for each of two meridian mapping runs. The order of the blocks was counterbalanced across runs and participants. Participants were asked to fixate and detect changes in the size of the fixation dot.

### Image Acquisition

Structural and functional magnetic resonance images were acquired through a 3T Philips Ingenia CX scanner with a 32-channel head coil at the Department of Radiology of the University Hospitals Leuven. A high-resolution structural scan was obtained through a T_1_-weighted three-dimensional turbo-field-echo sequence (182 slices, resolution 0.98 mm × 0.98 mm × 1.20 mm, TR = 9.60 ms, TE = 4.60 ms, 256 × 256 acquisition matrix). For task and meridian mapping runs, respectively, 209 and 251 whole-brain functional MRI volumes were acquired using a single-shot echo-planar imaging sequence (52 slices, resolution 2.50 mm × 2.50 mm × 2.50 mm, interslice gap 0.20 mm, TR = 1.50 s, TE = 30 ms, 96 × 96 acquisition matrix).

### Data Analysis

Data analyses were based on 24 out of 28 participants. One participant was excluded due to a congenital brain abnormality, another participant was excluded based on excessive head motion (> 1 × voxel size for translation and/or > 1 × voxel size for rotation) (Johnstone et al., 2006) and two participants did not complete the experiment due to technical issues in the MR data acquisition. The sample size of 24 participants was determined based on *a priori* power calculation (power = 0.80, effect size = 0.50, α = 0.05) with results of our previous study (Lanssens et al., 2020). Behavioral and eye tracking data were analyzed using R (R 3.5.3, R Core Team, 2021) and the lme4 package (lme4 1.1.21) (Bates et al., 2015). fMRI data processing and analysis was performed using Statistical Parametric Mapping (SPM12) software and custom-made scripts written in MATLAB (MATLAB 9.0, The MathWorks Inc., Natick, MA, United States).

#### Fixation Control

Eye tracking data were collected for fixation control during task runs using the Eyelink 1000 eye tracking system (1,000 Hz; SR research Ltd., 2018, Mississauga, ON, Canada). For the eye tracking data analyses, 11 out of 24 participants were excluded due to software issues that impeded the task and eye tracking data to be synchronized during the data acquisition. Additionally, 2 out of 24 participants were excluded based on eye tracking data not being successfully obtained in more than 2/3 task runs. The eye tracking data of all included participants (*N* = 11) were preprocessed by excluding task trials in which 30% or more of the samples were not successfully recorded during stimulus presentation. Data analysis consisted of calculating the proportion of trials for each task condition (attend to one or two streams of gratings) and each participant in which at least one saccade to at least one of the two stimuli occurred. Here, only eye movements with a minimal duration of 10 ms and an amplitude that fell within the respective x- and y-boundaries of the stimuli counted as saccades. Additionally, for the trials in which at least one saccade occurred, the average number of saccades per trial was calculated. Paired *t*-tests (α = 0.05) were performed to allow the pairwise comparison of the aforementioned variables between task conditions.

#### Behavior

To investigate the effect of attending to one or two streams of visual information, task accuracy was compared between respective conditions for go and no-go trials, as well as reaction times (RTs) for correct go trials. Trials with anticipatory RTs of 100 ms or less were excluded from the behavioral analysis. Accuracy levels on go and no-go trials were analyzed on a trial-by-trial level by applying a Poisson mixed-effects model with a random slope. On the other hand, RTs on correct go trials were analyzed on a trial-by-trial level by using a linear mixed-effects model with a random slope. Both the results on accuracy and RTs were presented as averaged values per participant.

#### fMRI

##### Preprocessing

The preprocessing stream for functional images of the feature-based go/no-go task used in the univariate region of interest (ROI) analysis was the same as for those used in the multivariate analysis. The first two functional images of each run in each participant were excluded from preprocessing and subsequent statistical analyses (Diedrichsen and Shadmehr, 2005). The preprocessing stream included motion correction by realigning functional images with the mean image within each run. Here, runs with excessive head motion (> 1 × voxel size for translation and/or > 1 × voxel size for rotation) (Johnstone et al., 2006) were excluded from the analyses. Motion correction was followed by co-registration of functional images with the anatomical (T_1_-weighted) image and spatial normalization into Montreal Neurological Institute (MNI) space while re-sampling to a voxel size of 3 mm × 3 mm × 3 mm (= 27 mm^3^). The functional images of task runs used in the univariate whole-brain analysis, as well as those of meridian mapping runs, were additionally preprocessed by performing spatial smoothing with an 8 mm full-width half-maximum kernel.

The preprocessing stream for functional images of the feature-based go/no-go task in the functional connectivity analysis included spatial normalization into MNI space, scrubbing, high-pass filtering above 0.01 Hz, regressing out white matter and ventricle signals, regressing out head motion, regressing out task-evoked activity using regressors of each of the respective conditions and low-pass filtering below 0.10 Hz. Here, the regressors of the task conditions were included to filter out statistical associations between timeseries of ROIs related to the timing of the task (Cole et al., 2019).

###### Regions of Interest

Multiple ROIs were defined for the univariate, multivariate and functional connectivity analyses (Table 1). Fronto-parietal ROIs (bilateral FEF, IPS and SPL) were created by thresholding and binarizing probabilistic maps (Wang et al., 2015). Here, the ROI for the IPS consisted of probabilistic maps of IPS1 and IPS2. In the visual cortex, bilateral V1-V4 were delineated in FreeSurfer (Martinos Center for Biomedical Imaging, Harvard-MIT, Boston, MA, United States). Each participant’s anatomical image was reconstructed in surface space and inflated, followed by a registration to the fsaverage-sym template space (Dale et al., 1999; Fischl et al., 1999; Greve et al., 2013). V1-V3 were defined based on a template by Benson et al. (2014). Since the template is less accurate near the foveal confluence and since the stimulus display was presented under 5° visual angle, the ROIs for V1-V3 were limited to an eccentricity of 1.50°–5° visual angle. On the other hand, V4 was delineated based on the meridian mapping task and anatomical landmarks. Specifically, V4 was defined as sharing the ventral border with V3 and extending up to the posterior transverse collateral sulcus (Witthoft et al., 2013). Here, the lower vertical meridian was used as the lateral border of V4, which was defined in each participant by constructing a general linear model (GLM) of the meridian mapping runs with two task regressors (one for each orientation of the wedges) and six motion regressors, followed by first-level contrasts between trials with horizontally and vertically oriented wedges. V1-V4 were converted from surface to volume space in the final step to construct the respective ROIs. Besides bilateral V1-V4, the bilateral lateral occipital cortex (LOC) and fusiform gyrus (FG) were included as ROIs in the visual cortex. These ROIs were defined by merging, thresholding and binarizing probabilistic maps from, respectively, Wang et al. (2015) and the Anatomy Toolbox in SPM12 software (Eickhoff et al., 2005).

**TABLE 1:** Bilateral regions of interest of the univariate, multivariate and functional connectivity analyses.

Type	Region	Number of voxels (27 mm^3^)
Probabilistic	FEF	790
	IPS	927
	SPL	431
	LOC	500
	FG	940
Individual	V1	290 (37)
	V2	284 (33)
	V3	470 (41)
	V4	270 (50)

*For each region of interest (ROI), the type (probabilistic or individually defined), anatomical region and number of voxels is reported. For probabilistic ROIs, the exact number of voxels is reported, while for individually defined ROIs, the mean and standard deviation of the number of voxels across participants is reported. Abbreviations: FEF = frontal eye fields, IPS = intraparietal sulcus, SPL = superior parietal lobe, LOC = lateral occipital cortex, FG = fusiform gyrus.*

###### Univariate Analysis

The GLM of the univariate ROI and whole-brain analyses of the feature-based go/no-go task contained two task regressors: one regressor for blocks of each condition (attend to one or two streams of gratings) with the duration set from cue onset to mask offset in the last trial of the block. Additionally, the GLM contained six motion regressors, one regressor for each translation and rotation in the three dimensions of space. After estimating the parameters of the GLM, in the univariate ROI analysis, first-level contrasts were defined for each condition vs. baseline by using a paired *t*-test. These first-level contrasts were combined in a group-level statistical analysis by estimating a random-effects model. Next, beta weights were extracted from the random-effects model for the bilateral fronto-parietal (FEF, IPS and SPL) and visual (V1-V4, LOC and FG) ROIs using the MarsBaR toolbox (Brett et al., 2002), followed by a paired *t*-test (α = 0.05) to compare the beta weights between task conditions. On the other hand, in the univariate whole-brain analysis, first-level contrasts between the task conditions were defined by using a paired *t*-test to determine whether activity in a respective voxel was stronger in one of both conditions, followed by a group-level statistical analysis with a random-effects model. The result of the univariate whole-brain analysis was presented with bspmview^1^ as a statistical parametric map relying on cluster-extent based thresholding, with the primary voxel-level threshold set to *p* < 0.001 and the secondary cluster-level threshold to *p* < 0.05 (corrected for multiple comparisons with the family wise error (FWE) method) (Poline et al., 1997).

To investigate whether activity levels in these block-level univariate analyses are biased by the cues, no-go trials and/or trials with mistakes, the result of the block-level whole-brain analysis was compared with that of an event-related (trial-by-trial) whole-brain analysis in which the correct go trials of each task condition were contrasted. This analysis was performed in the same way as the block-level whole-brain analysis, except that the GLM had five task regressors: two for the correct go trials of each condition (attend to one or two streams of gratings), one for all cues, one for all correct no-go trials and one for all trials with mistakes.

###### Multivariate Analysis

The multivariate analysis was performed using CoSMoMVPA (Oosterhof et al., 2016) and LIBSVM software (Chang and Lin, 2011) with custom-made scripts written in MATLAB (Ritchie and Op de Beeck, 2019). This analysis aimed at decoding whether one (magenta/cyan) or two streams of gratings were attended in blocks of the task for the bilateral fronto-parietal (FEF, IPS and SPL) and visual (V1-V4, LOC and FG) ROIs. The GLM of the multivariate analysis was similar to those of the univariate analyses, however, with separate regressors for blocks in which the magenta or cyan stream was attended (instead of one regressor for blocks in which one stream was attended). To obtain the *t*-statistics per run for the condition in which two streams were attended, the first-level contrast of the respective condition vs. baseline was calculated in the GLM. On the other hand, to obtain the *t*-statistics per run for the condition in which one stream was attended, the average first-level contrast of the magenta and cyan subconditions vs. baseline was calculated in the GLM. In this way, an equal amount of response patterns was obtained for the conditions (attend to one or two streams of gratings), which were used as input for a linear support vector machine (SVM). The cross-validation with the SVM was performed by training the classifier on a subset of response patterns of task runs (∼70%) to find the hyperplane that best separates the conditions where either one or two gratings were attended in each participant, followed by testing the classifier on the remaining task runs (∼30%). This k-fold cross-validation procedure (with the exact amount of folds depending on the number of included runs for the respective participant) was performed for several iterations until each possible combination of training and test set was acquired. The multivariate analysis resulted in one decoding accuracy averaged across folds and iterations for each participant and each ROI, reflecting the specificity of response patterns (thus, underlying neural representations) for different distributions of attention. Decoding accuracies were compared with chance-level performance (= half of the predictions correct, thus a decoding accuracy of 0.50) by using a paired *t*-test (α = 0.05).

###### Functional Connectivity Analysis

To investigate differences in the task-related interaction of fronto-parietal and visual ROIs between task conditions (attend to one or two streams of gratings), a functional connectivity analysis was performed using custom-made scripts written in MATLAB (Van Meel et al., 2019). Similar to the multivariate analysis, blocks in which the magenta and cyan stream were attended were (initially) analyzed separately. For each participant, timeseries of BOLD responses were extracted for each condition and averaged over voxels within a ROI. Functional connectivity was determined within participants by calculating the Pearson correlation between the BOLD timeseries of each pair of ROIs for each condition. Next, the Pearson correlation values of the magenta and cyan subconditions were averaged (into the condition in which one stream was attended), followed by converting all Pearson correlation values to Z-values by using the Fischer’s z-transformation. Group-level connectivity results for each task condition were produced by means of a one-sample *t*-test on the Z-values of individual participants. In addition, we obtained the contrast between conditions where either one or two gratings were attended by running a paired *t*-test on their respective Z-values. For each contrast, the significance level of α = 0.05 was corrected for multiple comparisons (i.e., number of pairwise connections) with the false discovery rate (FDR) method.

## Results

### Fixation Control

**FIGURE 2:**
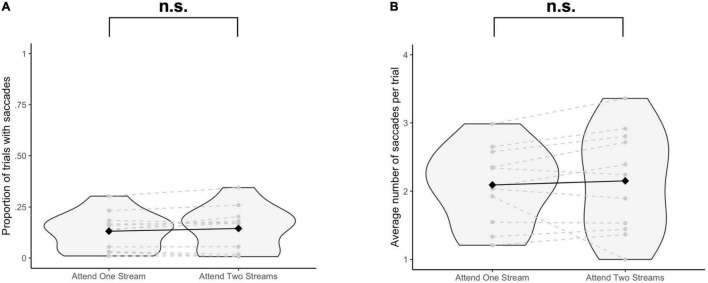
Results of the eye tracking data analysis. For each participant and each task condition, **(A)** the proportion of trials with at least one saccade (≥10 ms) to at least one of the stimulus positions is reported, and for the trials with saccades, **(B)** the average number of saccades per trial is reported. Significance was assessed by using a paired *t*-test. n.s., not significant (*p* > 0.05).

The proportion of trials with saccades was not significantly different when selecting one stream as opposed to attending to two streams of gratings [Δ = −0.01, 95% CI (−0.03, 0.005), *t*(10) = −1.64, *p* = 0.13] (Figure 2A). The average number of saccades per trial was also not significantly different when attending to one instead of two streams of gratings [Δ = −0.06, 95% CI (−0.31, 0.19), *t*(10) = −0.53, *p* = 0.61] (Figure 2B).

### Behavior

**FIGURE 3:**
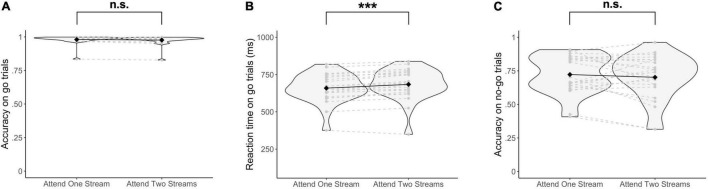
Behavioral results. **(A)** Accuracy on go trials, **(B)** Reaction times on go trials, and **(C)** Accuracy on no-go trials are reported. Significance was assessed by fitting a Poisson mixed-effects model on the trial-by-trial accuracy on go and no-go trials respectively, and a linear mixed-effects model on the trial-by-trial reaction times. ***p < 0.001, n.s., not significant (*p* > 0.05).

Behavioral data did not show a significant difference in accuracy on go trials when selecting one stream as opposed to attending to two streams of gratings [Δ = −0.004, 95% CI (−0.03, 0.02), *Z* = 0.33, *p* = 0.74] (Figure 3A). Similarly, no significant difference in the accuracy on no-go trials was found when attending to one as opposed to two streams of gratings [Δ = −0.03, 95% CI (−0.12, 0.05), *Z* = 0.83, *p* = 0.41] (Figure 3C). On the other hand, RTs were significantly increased when responding to two streams as opposed to selecting information of one stream of gratings [Δ = 25.03 ms, 95% CI (17.50 ms, 32.66 ms), *t*(22.67) = 6.49, *p* = 1.38 × 10^–6^] (Figure 3B).

### fMRI

#### Univariate Analysis

Beta weights (averaged across voxels) were significantly higher when selecting one stream instead of attending to two streams of gratings for the ROI corresponding to the bilateral SPL (Table 2). On the other hand, beta weights were significantly lower when attending to one as opposed to two streams of gratings for the ROI corresponding to the bilateral IPS and for the ROIs corresponding to bilateral V3, V4, LOC and FG.

**TABLE 2:** Univariate ROI analysis for the contrast of attending to one minus two streams of gratings.

	ROI	*t*(23)	*p*
Higher activity	SPL	**5.55**	**1.20 × 10** ^–^ **^5^**
	FEF	1.59	0.13
	V1	0.93	0.36
	V2	0.47	0.64
Lower activity	LOC	−**7.21**	**2.46 × 10** ^–^ **^7^**
	FG	−**5.28**	**2.35 × 10** ^–^ **^5^**
	V4	−**4.58**	**1.32 × 10** ^–^ **^4^**
	IPS	−**3.34**	**2.86 × 10** ^–^ **^3^**
	V3	−**3.10**	**5.01 × 10** ^–^ **^3^**

*In each participant, for each fronto-parietal and visual region of interest (ROI), beta weights (averaged across voxels) were extracted per task condition, followed by a paired t-test between respective task conditions. Significant p-values (α = .05) are indicated in bold.Abbreviations: FEF = frontal eye fields, IPS = intraparietal sulcus, SPL = superior parietal lobe, LOC = lateral occipital cortex, FG = fusiform gyrus.*

**FIGURE 4:**
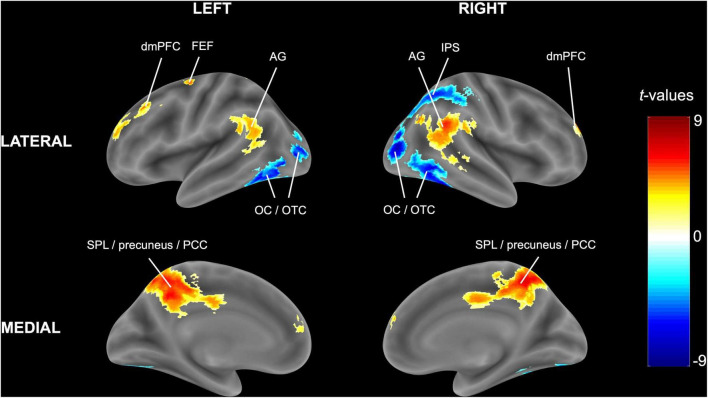
Univariate block-level whole-brain results. The statistical t-map in MNI (Montreal Neurological Institute) space is reported for the random-effects model of experimental blocks in which one stream of gratings was attended minus blocks in which two streams of gratings were attended. Cluster-extent based thresholding was used with voxel-based threshold *p* < 0.001 and cluster-based threshold FWE (familywise error)-corrected *p* < 0.05.

Congruent with the ROI analysis, in the (block-level) whole-brain analysis, attending to one stream instead of two streams of gratings resulted in higher activity in the bilateral SPL (extending into the precuneus and posterior cingulate cortex) and lower activity in the right IPS and bilateral occipital/occipitotemporal cortex (Figure 4 and Table 3). Additionally, increased activity in the left FEF, bilateral dorsomedial prefrontal cortex and bilateral angular gyrus was observed.

**TABLE 3:** Significant clusters in the univariate block-level whole-brain analysis for the contrast of attending to one minus two streams of gratings.

	Region	x, y, z (MNI)	Number of voxels (27 mm^3^)	*t(*23)	FWE-corr. *p*
Higher activity	Bilateral SPL + precuneus + posterior cingulate cortex	6, −52, 53	1260	7.19	< 0.001
	Right angular gyrus	57, −52, 26	406	6.39	< 0.001
	Bilateral dorsomedial prefrontal cortex	18, 53, 23	225	5.48	< 0.001
	Left FEF	−24, −13, 50	89	5.27	0.03
	Left angular gyrus	−57, −52, 20	149	4.43	0.003
Lower activity	Right occipital and occipitotemporal cortex + Right IPS	30, −79, 8	987	−8.87	< 0.001
	Left occipital and occipitotemporal cortex	−45, −61, −10	363	−7.26	< 0.001

*For each significantly activated cluster, the anatomical region, stereotactic MNI (Montreal Neurological Institute) coordinates, number of voxels, peak t-value and FWE (familywise error)-corrected p-value is reported. Cluster-extent based thresholding was used with primary threshold p < .001 and FWE-corrected p < .05. Abbreviations: SPL = superior parietal lobe, FEF = frontal eye fields, IPS = intraparietal sulcus.*

Results of the trial-by-trial whole-brain analysis on the correct go trials were congruent with those of the block-level whole-brain analysis (Supplementary Figure 1). Additionally, a trial-by-trial whole-brain analysis with the RTs on correct go trials set as trial durations in the GLM to correct for RT effects led to similar results (Supplementary Figure 2).

#### Multivariate Analysis

**FIGURE 5:**
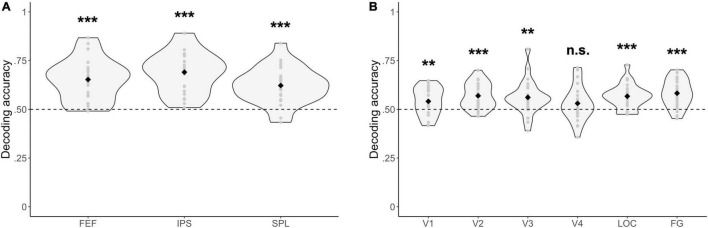
Multivariate fMRI results. Decoding accuracies are reported for **(A)** fronto-parietal, and **(B)** visual regions of interest between experimental blocks where either one or two streams of gratings were attended. Respective decoding accuracies were compared with chance-level performance (= dashed line) by using a paired *t*-test. ***p < 0.001, **p < 0.01, n.s., not significant (*p* > 0.05). FEF, frontal eye fields; IPS, intraparietal sulcus; SPL, superior parietal lobe; LOC, lateral occipital cortex; FG, fusiform gyrus.

For all fronto-parietal ROIs, decoding accuracies were significantly above chance when distinguishing between blocks where one stream had to be selected or two streams had to be attended [FEF: Δ = 0.15, 95% CI (0.11, 0.20), *t*(23) = 7.05, *p* = 3.49 × 10^–7^; IPS: Δ = 0.19, 95% CI (0.15, 0.23), *t*(23) = 8.86, *p* = 7.20 × 10^–9^; SPL: Δ = 0.12, 95% CI (0.08, 0.16), *t*(23) = 6.35, *p* = 1.75 × 10^–6^] (Figure 5A). Similarly, most visual ROIs (all, except for V4) had decoding accuracies that were significantly above chance when distinguishing between blocks where one as opposed to two streams of gratings had to be attended [V1: Δ = 0.04, 95% CI (0.01, 0.07), *t*(23) = 2.84, *p* = 9.24 × 10^–3^; V2: Δ = 0.07, 95% CI (0.04, 0.10), *t*(23) = 5.28, *p* = 2.35 × 10^–5^; V3: Δ = 0.06, 95% CI (0.02, 0.10), *t*(23) = 3.45, *p* = 2.16 × 10^–3^; V4: Δ = 0.03, 95% CI (−0.01, 0.07), *t*(23) = 1.68, *p* = 0.11; LOC: Δ = 0.07, 95% CI (0.04, 0.09), *t*(23) = 5.34, *p* = 2.00 × 10^–5^; FG: Δ = 0.08, 95% CI (0.05, 0.11), *t*(23) = 5.78, *p* = 6.91 × 10^–6^] (Figure 5B).

Considering the differences in the number of voxels between ROIs (Table 1), the multivariate analysis was also performed with a subset of 100 voxels in each ROI (defined as a sphere in the center of each ROI, with an equal distribution of voxels across hemispheres). Here, decoding accuracies were still significantly above chance for all fronto-parietal and most visual ROIs (all, except for V4) when distinguishing between blocks where either one or two streams of gratings had to be attended [FEF: Δ = 0.14, 95% CI (0.10, 0.18), *t*(23) = 7.75, *p* = 7.33 × 10^–8^; IPS: Δ = 0.14, 95% CI (0.11, 0.17), *t*(23) = 9.28, *p* = 3.08 × 10^–9^; SPL: Δ = 0.11, 95% CI (0.08, 0.15), *t*(23) = 6.34, *p* = 1.79 × 10^–6^; V1: Δ = 0.04, 95% CI (0.02, 0.07), *t*(23) = 3.47, *p* = 2.08 × 10^–3^; V2: Δ = 0.04, 95% CI (0.01, 0.07), *t*(23) = 2.55, *p* = 0.02; V3: Δ = 0.04, 95% CI (0.01, 0.06), *t*(23) = 3.19, *p* = 4.06 × 10^–3^; V4: Δ = 0.02, 95% CI (−0.01, 0.05), *t*(23) = 1.60, *p* = 0.12; LOC: Δ = 0.04, 95% CI (0.01, 0.06), *t*(23) = 3.12, *p* = 4.76 × 10^–3^; FG: Δ = 0.04, 95% CI (0.01, 0.06), *t*(23) = 2.93, *p* = 7.47 × 10^–3^].

#### Functional Connectivity Analysis

**FIGURE 6:**
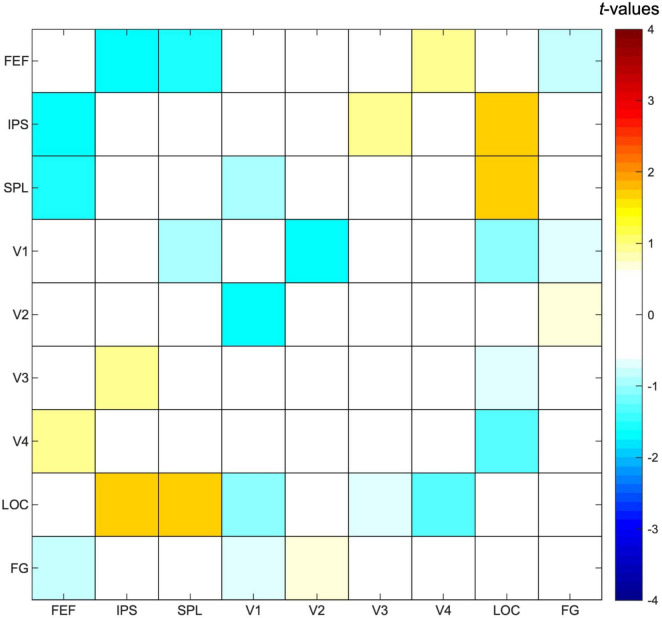
Functional connectivity results. Pearson correlation values were calculated between timeseries of fronto-parietal and visual regions of interest for each participant and task condition, followed by a paired *t*-test to contrast the conditions in which either one or two streams of gratings were attended. Results are presented as *t*-values, in which significance was assessed by comparing with a FDR (false discovery rate)-corrected threshold equal to *q* < 0.05 to correct for multiple comparisons. FEF, frontal eye fields; IPS, intraparietal sulcus; SPL, superior parietal lobe; LOC, lateral occipital cortex; FG, fusiform gyrus.

Selecting one stream compared to attending to two streams of gratings did not result in any significant differences in functional connectivity between ROIs (Figure 6), although we observed a trend for increased functional connectivity between parietal and visual ROIs when attending to one instead of two streams of gratings, specifically between ROIs corresponding to the bilateral IPS and LOC [*t*(23) = 1.67, *p* = 0.05] and the bilateral SPL and LOC [*t*(23) = 1.63, *p* = 0.06].

## Discussion

The current study aimed to investigate whether activity in subregions of the DAN and the visual cortex is modulated by feature-based attentional weighting, and if so, whether their timecourses are correlated. Results of both the univariate ROI and whole-brain analysis showed that activity levels for subregions of the DAN and visual cortex were modulated by attending to one as opposed to two streams of gratings, with higher activity levels in the SPL and lower activity levels in the IPS and occipital/occipitotemporal regions (Figure 4 and Tables 2, 3) across the analyses. The result of a trial-by-trial whole-brain analysis on the correct go trials was almost identical, which suggests that results of these block-level univariate analyses were not biased by the inclusion of cues, no-go trials and/or incorrect trials (Supplementary Figure 1). The multivariate analysis further demonstrated that activity patterns in all subregions of the DAN (bilateral FEF, IPS and SPL) (Figure 5A), as well as those in multiple visual regions (bilateral V1-V3, LOC and FG) (Figure 5B), are modulated by feature-based attentional weighting. The functional connectivity analysis did not reveal significantly increased functional connectivity between subregions of the DAN and the visual cortex when selecting one instead of attending to two streams of gratings (Figure 6).

### Activity in Visual Regions

Prioritizing one or two streams of gratings based on color led to different activity levels in several regions in the visual cortex. In the univariate ROI analysis, bilateral visual regions (V3-V4, LOC and FG) had decreased activity when attending to one instead of two streams of gratings. This is congruent with results of the univariate whole-brain analysis, where a decrease in activity for bilateral visual regions (mostly corresponding to FG) was observed. Since the stimulus display was sensorially matched between task conditions, these results can be attributed to a top-down modulation of stimulus representations in the visual cortex (e.g., Kastner and Ungerleider, 2000; Maunsell and Treue, 2006) and are unlikely to be caused by bottom-up mechanisms. The current results seem to be consistent with those of previous studies on top-down processes in the visual cortex, where a classifier was trained to dissociate activity patterns of multiple physical colors and subsequently tested on, for instance, ambiguous colors or word cues (Brouwer and Heeger, 2009; Vandenbroucke et al., 2014; Zhang et al., 2018). Results suggested that top-down processes target intermediate- (V3/V4) and high-order (LOC) visual regions. However, several studies found that an attended feature value (e.g., a direction of motion) can be decoded in early visual areas as well (Kamitani and Tong, 2006; Serences et al., 2009). This can be explained by studies suggesting that top-down signals propagate from high- to low-order visual regions, by which top-down effects are larger and earlier in the high-order visual areas (Martinez et al., 1999; Buffalo et al., 2010). Therefore, a multivariate analysis was useful, since it is a more sensitive tool to detect differences in activity of ROIs between task conditions than a univariate analysis as it takes into account all activations (not averaged with those of surrounding voxels) to consider spatial patterns associated with task conditions (Norman et al., 2006). Results of the multivariate analysis indeed revealed that not only activity patterns of high-order, but also those of low-order visual regions (bilateral V1-V3, LOC and FG) are modulated by feature-based attentional weighting. However, decoding accuracies were not significantly different from chance-level for the ROI corresponding to V4. This is unlikely to be related to the more restricted size of the ROI (Table 1), since a multivariate analysis with an equal amount of voxels across ROIs (*N* = 100) did not lead to substantially different findings. Thus, attention-related effects in activity patterns of the color-sensitive region V4 were most likely too small to be detected by the classifier when dissociating between conditions in which feature values were identical.

Noteworthy, congruent with results of the univariate analyses, decoding accuracies in the multivariate analysis were most reliable on the level of high-order visual regions. Several neuroimaging studies have showed that different perceptual representations of objects (i.e., global vs. local) modulate activity levels in the high-order visual cortex (Murray et al., 2002; Kourtzi et al., 2003; Stoll et al., 2020). Therefore, processes related to object recognition and processing might (partially) attribute to findings on the level of the high-order visual regions LOC and FG (Bar et al., 2001; Grill-Spector et al., 2001; Kourtzi and Kanwisher, 2001), where attending to two streams of gratings might have led to a different perceptual representation of the stimulus display compared to selecting only one stream of gratings (e.g., as one instead of separate objects).

### Activity in Fronto-Parietal Dorsal Attention Regions

Not only activity in visual regions, but also activity in regions of the DAN was modulated by the distribution of attention. The multivariate analysis showed that activity patterns in all subregions of the DAN (bilateral FEF, IPS and SPL) are modulated by feature-based attentional weighting. Congruently, the univariate ROI and whole-brain analysis demonstrated differences in activity levels for regions of the DAN between task conditions. Specifically, bilateral SPL activity was higher when attending to one instead of two streams of gratings. This could be related to the involvement of the SPL in shifting attention (Vandenberghe et al., 2001; Yantis et al., 2002; Molenberghs et al., 2007), where the SPL uses information on attentional priority to re-orient attention toward the location of the relevant grating in blocks where one stream was selected, which was not the case in blocks where both gratings required attention. On the other hand, decreased activity levels in the (right) IPS were observed when attending to one as opposed to two streams of gratings. This finding is seemingly incongruent with its role in selective attention, but congruent with several studies on working memory which report a correlation of IPS activity with working memory load (Cohen et al., 1997; Todd and Marois, 2004; Xu and Chun, 2006, 2009; Gillebert et al., 2012b). Since relevant stimuli are benefited over distracters in gaining access to working memory (Bundesen, 1990), it is likely that the working memory load was different between task conditions. Furthermore, similar to aforementioned results on the level of high-order visual regions, the current finding can also be seen from the perspective of object perception since study-based evidence in cognitively healthy (Zaretskaya et al., 2013; Grassi et al., 2016) and clinal (Karnath et al., 2000; Huberle and Karnath, 2006) populations suggests that the parietal cortex mediates global perceptual processing. This finding does, however, not contradict a possible involvement of the IPS in selective attention, given that attention and working memory (Gazzaley, 2011; Lepsien et al., 2011), as well as attention and perception (Gillebert et al., 2016) are related cognitive processes, and that regions of the DAN support multiple cognitive processes. Further research on how the IPS can support multiple processes in parallel could be valuable. One possible explanation is the existence of a functional dissociation within the IPS, where the inferior subregion selects objects that gain access to memory (consistent with a role in selective attention), while the superior subregion encodes the features of (a subset of) the selected objects (consistent with a role in working memory) (Xu and Chun, 2006, 2009). Further, the univariate whole-brain analysis indicated increased activity in the left FEF when attending to one as opposed to two streams of gratings. This difference in activity was not observed for the ROI corresponding to the bilateral FEF in the univariate ROI analysis. The FEF could contain a representation of attentional priority to plan and execute saccades to the selected item in the visual field (Bruce and Goldberg, 1985; Bichot and Schall, 1999; Schall and Thompson, 1999). In the current study, this finding would be related to planning rather than executing eye movements since the eye tracking data analysis did not reveal any significant differences in the proportions of trials with at least one saccade and the average number of saccades per trial between task conditions (Figure 2).

A possible confound for results on the level of fronto-parietal regions that needs to be addressed is a difference in difficulty level between task conditions. Considering that task difficulty modulates activity in fronto-parietal dorsal attention regions (e.g., Duncan, 2010; Fedorenko et al., 2013) and since the behavioral results of the current study suggest that responding to two instead of one streams of gratings was more difficult (Figure 3), this might attribute to the observed differences in fronto-parietal activity between task conditions. However, since a trial-by-trial whole-brain analysis with RTs on correct go trials as trial durations had a similar result (Supplementary Figure 2) as the block-level whole-brain analysis (Figure 4) and the aforementioned trial-by-trial whole-brain analysis (Supplementary Figure 1), it is unlikely that differences in effects related to RT (e.g., task difficulty) can explain observations of the current study (Grinband et al., 2008; Woolgar et al., 2014).

### Functional Interactions Between Fronto-Parietal and Visual Regions

The functional connectivity analysis revealed that functional connectivity was not significantly different between fronto-parietal and visual regions when attending to one as opposed to two streams of gratings. A trend toward significantly increased functional connectivity between bilateral IPS and LOC, as well as between bilateral SPL and LOC, can be observed that is unlikely to be related to object perception, because the involvement of parietal regions in perceptual processes is related to global rather than local processing (e.g., Karnath et al., 2000; Zaretskaya et al., 2013). Therefore, this could be the result of top-down signals from parietal subregions of the DAN to the high-order visual cortex based on attentional priority. However, since the current analysis did not result in significant findings and taking into account that it cannot infer causality, further research is necessary to support this claim. An explanation on why the current study did not find significant differences in functional connectivity between task conditions in any of the ROIs is that the between-subject variability in functional connectivity was relatively high. For the ROIs with the most different functional connectivity between task conditions (IPS and LOC), a power calculation showed that around 170 participants would be needed for this analysis to have sufficient statistical power (power = 0.80, effect size = 0.30, α = 0.05). We argue that increasing the sample size would most likely result in significant differences between task conditions for the aforementioned ROIs.

While most evidence on a top-down role of the DAN in selective attention centers around its parietal subregions, several studies argue that top-down signals originate in the FEF (Zhou and Desimone, 2011; Ibos and Freedman, 2014; Bichot et al., 2015; Yin and Liu, 2018). Here, study-based evidence suggests that both FEF and IPS contain a representation of attentional priority, but that the FEF modulate both the IPS and visual regions in the process of selective attention (Bressler et al., 2008; Sapountzis et al., 2018). Furthermore, in feature-based attention, studies by Zanto et al. (2010, 2011) suggest that a region outside the DAN, the inferior frontal junction (IFJ), might be the ultimate top-down source. This is corroborated by recent fMRI studies, which used multiple analysis techniques (e.g., multivariate analysis, dynamic causal modeling, Granger causality) to demonstrate that the IFJ influences activity in regions of the DAN and in the visual cortex (Zhang et al., 2018; Meyyappan et al., 2021). Thus, future studies need to focus on investigating interactions within the DAN and between regions of the DAN and other regions (e.g., the IFJ) to identify the critical nodes involved in (different top-down mechanisms of) selective attention, which will aid the development of a more complete account of attention and its related disorders.

### Interaction Between Spatial- and Feature-Based Attentional Weighting

The go/no-go task used in the current study relied on feature-based attentional weighting, where the features (and not the spatial locations) of the stimuli determined their behavioral relevance. However, while feature-based attentional weighting was required to identify the relevant stimulus, this process was most likely followed by spatial mechanisms to shift attention toward and to enhance stimulus processing at its respective location. It is important to note that the spatial component is much smaller than the feature component in the task of the current study since the stimuli were presented in close proximity at the fovea. Furthermore, an interaction of spatial- and feature-based attention regularly occurs in real-life situations and is inherent to the way in which visual attention is organized, that is through topographic maps of the environment which represent the attentional priority of items in the visual field based on their bottom-up and top-down values (Koch and Ullman, 1985; Itti and Koch, 2001; Bisley and Mirpour, 2019). Thus, enhancing a task-relevant feature (value) results in increased attentional priority for items with that feature (value) at their respective locations in the topographic map, by which items at these locations are preferentially attended and processed. Noteworthy, the spatial locations that were attended and processed were the same across task conditions of the current study considering that stimuli randomly switched positions from trial to trial and that analyses were performed on the block-level. Therefore, the task conditions were matched in terms of spatial mechanisms of selective attention and only differed in that feature-based attentional weighting was required when one as opposed to two streams of gratings had to be attended. As a future perspective, it would be valuable to combine the current paradigm with techniques providing a better temporal resolution, such as EEG or MEG, to further investigate the interaction between the closely linked processes of spatial- and feature-based attention (Wildegger et al., 2017).

### Conclusion

The current study demonstrates that activity in all regions of the DAN, as well as activity in low- to high-order visual regions, is modified by feature-based attentional weighting. Further research is required to elucidate whether subregions of the DAN, either directly or indirectly, have a top-down role in modifying stimulus representations in the visual cortex based on feature priority.

## Data Availability Statement

The datasets presented in this article are not readily available because the ethics approval does not permit the public archiving of data supporting the current study, and sharing of data requires a formal data-sharing agreement in accordance with ethical procedures governing the re-use of sensitive data. Requests to access the datasets should be directed to the corresponding author CG: celine.gillebert@kuleuven.be.

## Ethics Statement

The studies involving human participants were reviewed and approved by Ethics Committee Research UZ/KU Leuven. The patients/participants provided their written informed consent to participate in this study. Written informed consent was obtained from the individual(s) for the publication of any potentially identifiable images or data included in this article.

## Author Contributions

AL was responsible for the data collection and for drafting the manuscript. All authors contributed to the design and data analysis of the current study, as well as to the revision the manuscript, and approved the final version of the manuscript for publication.

## Conflict of Interest

The authors declare that the research was conducted in the absence of any commercial or financial relationships that could be construed as a potential conflict of interest.

## Publisher’s Note

All claims expressed in this article are solely those of the authors and do not necessarily represent those of their affiliated organizations, or those of the publisher, the editors and the reviewers. Any product that may be evaluated in this article, or claim that may be made by its manufacturer, is not guaranteed or endorsed by the publisher.

## Footnotes

1
http://www.bobspunt.com/bspmview

## Supplementary Material

Supplementary Figure 1 Univariate trial-by-trial whole-brain results. The statistical t-map in MNI (Montreal Neurological Institute) space is reported for the random-effects model of correct go trials in which one stream of gratings was attended minus correct go trials in which two streams of gratings were attended. Cluster-extent based thresholding was used with voxel-based threshold *p* < 0.001 and cluster-based threshold FWE (familywise error)-corrected *p* < 0.05.

Supplementary Figure 2 Univariate trial-by-trial whole-brain results with reaction times as trial durations. The statistical t-map in MNI (Montreal Neurological Institute) space is reported for the random-effects model of correct go trials in which one stream of gratings was attended minus correct go trials in which two streams of gratings were attended, with the respective reaction times on go trials as trial durations to account for reaction time effects. Cluster-extent based thresholding was used with voxel-based threshold *p* < 0.001 and cluster-based threshold FWE (familywise error)-corrected *p* < 0.05.
